# Temporal relationships between incarceration and mental disorders among justice-involved adolescents: A population-based cohort study

**DOI:** 10.1177/10398562251383801

**Published:** 2025-10-03

**Authors:** Emaediong I Akpanekpo, Azar Kariminia, Preeyaporn Srasuebkul, Julian N Trollor, John Kasinathan, David Greenberg, Tony Butler

**Affiliations:** School of Population Health, 7800 UNSW Sydney, Sydney, NSW, Australia; The Kirby Institute, 7800 UNSW Sydney, Sydney, NSW, Australia; National Centre of Excellence in Intellectual Disability Health, Faculty of Medicine and Health, 7800 UNSW Sydney, Sydney, NSW, Australia; National Centre of Excellence in Intellectual Disability Health, Faculty of Medicine and Health, 7800 UNSW Sydney, Sydney, NSW, Australia; Centre for Healthy Brain Ageing, Faculty of Medicine and Health, 7800 UNSW Sydney, Sydney, NSW, Australia; 161777 Justice Health and Forensic Mental Health Network, Sydney, NSW, Australia; School of Population Health, 7800 UNSW Sydney, Sydney, NSW, Australia

**Keywords:** adolescents, mental disorders, custody, incarceration, youth justice

## Abstract

**Objectives:**

To determine bidirectional relationships between incarceration and mental disorders. We hypothesized that (1) pre-existing mental disorders would be associated with increased incarceration risk, and (2) incarceration would be associated with increased incident mental disorder diagnosis risk in adolescents without prior psychiatric diagnosis.

**Method:**

This retrospective cohort study included 1551 adolescents (aged 10–17 years) from four New South Wales (NSW) health surveys linked to justice and health records. Modified Poisson regression examined associations between pre-existing mental disorders and incarceration. Prentice-Williams-Peterson Total-Time models examined associations between time-varying incarceration exposure and incident mental disorder diagnoses among those without prior diagnosis.

**Results:**

Among 1551 adolescents (87.7% male; median age 15 years), pre-existing mental disorders were associated with an increased incarceration risk (adjusted Risk Ratio [RR]: 1.26, 95% Confidence Interval [CI]: 1.09–1.45), which was more pronounced among those with violent offences. Among 1424 adolescents without prior diagnosis, incarceration was associated with increased incident diagnosis risk (adjusted Hazard Ratio [aHR]: 1.22, 95% CI: 1.09–1.37), with stronger associations among adolescents residing in areas of higher socioeconomic disadvantage.

**Conclusions:**

Findings suggest incarceration may be associated with adverse mental health outcomes, with implications for diversionary pathways and custodial mental health care.

Globally, incarcerated adolescents have a disproportionate burden of mental disorders compared to their peers in the general population and Australia is no exception, with its incarcerated young people showing a similarly high prevalence of these conditions.^1–3^ Pooled prevalence estimates of mental disorders are as high as 81%,^3^ a figure compounded by comorbidity.^4,5^ The high prevalence of mental ill-health among incarcerated adolescents raises a question about the nature and direction of this association. Establishing causality would require the random assignment to incarceration, which is ethically and practically impossible. Evidence linking incarceration exposure to mental health outcomes has largely relied on observational data.

A key limitation of existing studies is that temporal order is yet to be established. Most studies include justice-involved adolescents with pre-existing mental disorders, making it challenging to disentangle whether subsequent mental health outcomes reflect the progression of an existing condition or are potentially attributable to the incarceration experience. A prior study, for example, showed that both the prevalence and incidence of psychiatric hospitalizations among justice-involved adolescents increased with the severity of their justice system involvement.^6^ Like studies documenting similar phenomena in adult prisoners,^7^ this may be indicative of increased service use from referrals to mental health services,^8^ a reflection of the worsening of a pre-existing condition due to the stress of re-entry,^9^ a delayed clinical response to a disorder that had its onset during incarceration, or the emergence of a new disorder directly precipitated by the incarceration experience. Without clear temporal separation between the onset or diagnosis of mental disorder and the incarceration event, these competing explanations cannot be disentangled.

To address this gap, we investigated the temporal relationships between incarceration and mental health disorders among justice-involved adolescents using a bidirectional approach. First, we examined whether pre-existing mental disorders increased the risk of subsequent incarceration. Second, we examined whether incarceration increased the risk of incident mental disorder diagnoses among adolescents with no prior psychiatric history. We hypothesized that both relationships would be present: pre-existing mental disorders would be associated with an increased incarceration risk, and incarceration would be associated with an increased risk of incident mental disorder diagnoses.

## Methods

### Study design and population

We conducted a retrospective cohort study using linked administrative and health survey data in New South Wales (NSW), Australia. The study population comprised 1551 adolescents (aged 10–17 years at index offence) involved with the NSW youth justice system (either in custody or serving community orders) who participated in one of four health surveys conducted intermittently between 2003 and 2015: (1) the 2003 Young People in Custody Health Survey (YPiCHS), (2) the 2003–2006 Young People on Community Orders Health Survey (YPoCOHS), (3) the 2009 YPiCHS, and (4) the 2015 YPiCHS.^10–13^ All four surveys employed a total population sampling method.

To form the cohort, we linked to five data collections. The data collections included the NSW Bureau of Crime Statistics and Research’s (BOCSAR) Reoffending Database (ROD), the NSW Admitted Patient Data Collection (APDC), the NSW Mental Health Ambulatory Data Collection (MH-AMB), the NSW Emergency Department Data Collection (EDDC), and the NSW Registry of Births, Deaths, and Marriages (RBDM). Data linkage was performed by the Centre for Health Record Linkage (CHeReL) using probabilistic matching algorithms. Details of information contained in these data sources are presented in the supplemental material. Timeline of data source availability is illustrated in Supplemental Figure 1. The cohort selection process is detailed in Supplemental Figure 2.

### Ethics approval

Ethical approval was obtained from the NSW Population & Health Services Research Ethics Committee (Approval No. 2019/ETH13028), the Human Research Ethics Committee of the Justice Health and Forensic Mental Health Network (Approval No. G692/15), and the Aboriginal Health & Medical Research Council Ethics Committee (Approval No. 1394/18). The study was conducted in accordance with the Declaration of Helsinki. Participants provided informed consent for data linkage at the time of the original health surveys. For the present secondary analysis of de-identified linked data, a waiver of the requirement for consent was granted by the NSW Population & Health Services Research Ethics Committee.

### Exposure

The primary exposure was incarceration in a NSW Youth Justice Centre, ascertained from the ROD (Supplemental Table 1). Exposure time for each incarceration episode included any contiguous period of remand preceding court finalization. We also characterized incarceration using: (a) cumulative duration of incarceration (accrued prior to each risk interval), and (b) cumulative count of incarceration episodes.

### Outcome

The primary outcome was incident mental health disorder diagnoses ascertained during the follow-up period (from the first proven offence date up to the day before the 20th birthday). Incident events were defined as new diagnoses recorded on different dates or during distinct service episodes, with a minimum of 30 days required between diagnoses within the same diagnostic category. These diagnoses were identified using International Statistical Classification of Diseases and Related Health Problems, Tenth Revision, Australian Modification (ICD-10-AM) codes recorded in the APDC, MH-AMB, and EDDC datasets. EDDC presentations coded using the Systematized Nomenclature of Medicine – Clinical Terms (SNOMED-CT) were mapped to their equivalent ICD-10-AM classifications.

Mental health disorder diagnoses were categorized into psychosis, mood disorders, anxiety disorders, personality disorders, substance use disorders, and behavioural disorders. Detailed codes for each category are presented in Supplemental Table 2. Although the ICD-10-AM codes used for ascertainment were mutually exclusive, participants could be classified into multiple diagnostic categories if they received diagnoses across different categories. However, consistent with previous research,^2,14^ a hierarchical method was applied to create mutually exclusive groups for the psychosis, mood, and anxiety disorder categories. Therefore, if a young person had recorded diagnoses for both a psychotic disorder and a mood or anxiety disorder, they were classified only in the psychotic disorders category.

### Covariates

Covariates for all multivariable models were selected based on a review of the existing literature on risk factors for adolescent/youth incarceration and poor mental health.^2,3,5,6,14–18^ These variables included age, sex, Indigenous status, index offence type, area-level socioeconomic disadvantage, parental incarceration, parental death, out-of-home care history and history of head injury. Birth cohort was included to account for cohort effects. Details of covariate definitions and operationalization are presented in Supplemental Table 1.

### Statistical analysis

Descriptive statistics (counts [*N*], percentages [%], median, and inter-quartile range [IQR]) were used to summarize baseline characteristics and justice system involvement during follow-up, both overall and stratified by pre-existing mental health disorder status. Further descriptive analyses characterized the proportion of incident mental health diagnoses.

To examine the association between pre-existing mental disorders and the risk of subsequent incarceration, we estimated Risk Ratios (RRs) using modified Poisson regression with a log link and robust error variances. The multivariable model adjusted for the full set of selected covariates, all of which were fixed at baseline and were retained regardless of statistical significance. An additional model included an interaction term between pre-existing mental disorder status and index offence type.

To examine the association between incarceration and incident mental disorders, we analysed 1424 adolescents after excluding those with pre-existing mental health disorders (*n* = 127). We used a Prentice-Williams-Peterson Total-Time (PWP-TT) approach to model recurrent events,^19^ an extension of the Cox proportional hazards model. Details on model specifications for the PWP-TT model are available in the supplemental material. The PWP-TT model estimated the adjusted hazard ratio (aHR) for incarceration, adjusted for the full set of covariates. Additional models examined interactions with area-level socioeconomic disadvantage and alternative characterizations of incarceration. Cluster-robust standard errors were used to account for multiple events per participant.

Sensitivity analyses were conducted to assess the robustness of the primary findings. First, we repeated the analysis excluding participants with substance use disorders and substance-induced psychosis to examine whether the primary finding was disproportionately driven by the potentially confounding bidirectional relationship between substance use and justice system involvement. Second, to address potential reverse causation and detection bias, we performed lag-time analyses ignoring events occurring within the first 6 months (and separately, 1 year) following initiation of the first incarceration episode. Finally, we calculated an E-value to quantify the potential impact of unmeasured confounding.

Little’s test supported the missing completely at random (MCAR) assumption (p > .05). Given the minimal count of missing data (see Supplemental Figure 2) and the supported MCAR assumption, complete case analysis was employed. Statistical significance for all analyses was set at p < .05. Data preparation was conducted using SAS version 9.4 (SAS Institute, Cary, NC) and analyses performed using Stata version 19.5 (StataCorp, College Station, TX).

## Results

### Characteristics of the study population

The study included 1551 justice-involved adolescents (87.7% male; median age at first offence: 15 years [IQR: 14–16]), with 37.2% residing in the most socioeconomically disadvantaged areas (Table 1). Participants with pre-existing mental disorders (*n* = 127, 8.2%) differed from those without in several characteristics: a higher proportion were female (22.0% vs 11.4%), had experienced parental incarceration (33.1% vs 25.5%), and had prior out-of-home care placements (27.6% vs 15.9%). During follow-up, participants experienced multiple convictions on average (median 4 [IQR: 2–6]). About half (51.8%) experienced both community and custody sentences (Table 2).

**Table 1.** Baseline characteristics of the study population (*N* = 1551)

Characteristics	Overall *N* = 1551	No pre-existing mental disorder *N* = 1424	Pre-existing mental disorder *N* = 127
Age at first offence (years)			
Median (IQR)	15 (14, 16)	15 (14, 16)	15 (14, 16)
Mean (SD)	15.1 (1.8)	15.1 (1.8)	15.2 (1.8)
Sex			
Female	191 (12.3)	163 (11.4)	28 (22.0)
Male	1360 (87.7)	1261 (88.6)	99 (78.0)
Indigenous status			
Indigenous	536 (34.6)	486 (34.1)	50 (39.4)
Non-indigenous	1015 (65.4)	938 (65.9)	77 (60.6)
Socioeconomic disadvantage^a^			
Q1 (most disadvantaged)	577 (37.2)	543 (38.1)	34 (26.8)
Q2	369 (23.8)	336 (23.6)	33 (26.0)
Q3	376 (24.2)	340 (23.9)	36 (28.3)
Q4 (least disadvantaged)	157 (10.1)	139 (9.8)	18 (14.2)
Missing	72 (4.6)	66 (4.6)	6 (4.7)
Parental incarceration			
≥1 parent	405 (26.1)	363 (25.5)	42 (33.1)
Parental death			
≥1 parent	126 (8.1)	117 (8.2)	9 (7.1)
Out-of-home care			
≥1 placement	262 (16.9)	227 (15.9)	35 (27.6)
Head injury			
Yes	455 (29.4)	419 (29.4)	36 (28.3)
Index offence			
Violent	720 (46.4)	663 (46.6)	57 (44.9)
Non-violent	831 (53.6)	761 (53.4)	70 (55.1)
Number of concurrent offences at index offence			
Median (IQR)	2 (1, 4)	2 (1, 4)	3 (1, 5)
Mean (SD)	3.3 (3.2)	3.2 (3.1)	4.5 (4.2)

Baseline is the date of first proven offence. IQR – Inter-Quartile Range; SD – Standard Deviation.

^a^Socioeconomic disadvantage was assessed using the Index for Relative Socioeconomic Disadvantage (IRSD) quartiles from the Australian Bureau of Statistics’ Socio-Economic Indexes for Areas (SEIFA).

**Table 2.** Justice system involvement characteristics during follow-up (*N* = 1551)

Characteristics	Overall *N* = 1551	No pre-existing mental disorder *N* = 1424	Pre-existing mental disorder *N* = 127
Follow-up period (years)			
Median (IQR)	4 (3, 5)	4 (3, 5)	4 (2, 5)
Number of convictions			
Median (IQR)	4 (2, 6)	4 (2, 6)	3 (1, 7)
Mean (SD)	4.5 (3.5)	4.5 (3.4)	4.4 (3.5)
Number of custodial sentences			
Median (IQR)	1 (0, 3)	1 (0, 3)	1 (0, 3)
Mean (SD)	1.9 (2.5)	1.9 (2.5)	2.0 (2.4)
Number of community sentences			
Median (IQR)	2 (1, 3)	2 (1, 3)	2 (1, 3)
Mean (SD)	2.4 (1.9)	2.4 (1.9)	2.4 (2.2)
Sentence exposure pattern, *n* (%)			
Both community and custody^a^	804 (51.8)	738 (51.8)	66 (52.0)
Custody sentence only	171 (11.0)	153 (10.7)	18 (14.2)
Community sentence only	508 (32.8)	471 (33.1)	37 (29.1)
Others	68 (4.4)	62 (4.4)	6 (4.7)
Total custodial duration (months)			
Median (IQR)	12 (0, 42)	12 (0, 42)	14 (0, 42)
Mean (SD)	27.3 (37.3)	27.1 (36.8)	29.8 (42.3)
Total community sentence duration (months)			
Median (IQR)	24 (12, 42)	24 (12, 42)	24 (9, 42)
Mean (SD)	28.3 (24.4)	28.3 (24.4)	27.5 (25.4)

IQR – Inter-quartile Range; SD – Standard Deviation.

^a^Both community and custody’ indicates individuals experienced at least one episode of each type during follow-up.

### Association between pre-existing mental health disorders and incarceration

In the multivariable analysis (*N* = 1479), pre-existing mental health disorders were associated with an increased risk of incarceration (adjusted RR, 1.26; 95% CI, 1.09–1.45; Supplemental Table 3). The E-value of 1.83 (lower CI: 1.40) suggests moderate robustness to unmeasured confounding. However, this risk was modified by the type of index offence (Supplemental Table 4). A violent index offence significantly increased the risk of incarceration among adolescents with a pre-existing mental disorder (adjusted RR, 1.48; 95% CI, 1.11–1.97). Formal statistical testing confirmed a significant positive interaction on both the multiplicative scale (ratio of adjusted RRs = 1.57; 95% CI, 1.18–2.08) and the additive scale. On the additive scale, the interaction accounted for 37% of the total risk among those with both factors present (Attributable Proportion; 95% CI, 19%–54%).

### Incidence proportion of mental disorder diagnosis

The analytic sample for incident disorders included 1424 adolescents with a median follow-up duration of 4 years (IQR: 3–5). Overall, nearly half of the cohort (47.1%, *n* = 670) received at least one incident mental disorder diagnosis during follow-up (Table 3). This proportion was higher among participants who experienced incarceration (55.2%) compared to those who did not (38.3%) (Table 4). Of all participants with an incident diagnosis, 14.0% (*n* = 94) received diagnoses across multiple categories.

**Table 3.** Proportion of justice-involved adolescents diagnosed with mental disorders during follow-up, overall and stratified by sex and age groups (*N* = 1424)

Mental health disorder	Total (*N* = 1424) *N* (%)	Female (*n* = 163) *n* (%)	Male (*n* = 1261) *n* (%)	Age 10–13 (*n* = 263) *n* (%)	Age 14–17 (*n* = 1161) *n* (%)
Any mental disorder	670 (47.1)	96 (58.9)	574 (45.5)	186 (70.7)	484 (41.7)
Psychosis	73 (5.1)	8 (4.9)	65 (5.2)	22 (8.4)	51 (4.4)
Mood disorders	110 (7.7)	24 (14.7)	86 (6.8)	33 (12.6)	77 (6.6)
Anxiety disorders	77 (5.4)	23 (14.1)	54 (4.3)	18 (6.8)	59 (5.1)
Personality disorders	50 (3.5)	12 (7.4)	38 (3.0)	19 (7.2)	31 (2.7)
Substance use disorder	330 (23.2)	55 (33.7)	275 (21.8)	93 (35.4)	237 (20.4)
Behavioural disorders	96 (6.7)	8 (4.9)	88 (7.0)	36 (13.7)	60 (5.2)

Any mental disorder refers to any of the diagnostic categories (psychosis, mood disorders, anxiety disorders, personality disorders, substance use disorders, or behavioural disorders).

**Table 4.** Association between incarceration and incident recurrent mental disorder diagnoses (*N* = 1358)

Analysis scenario	Participants	Total events	Participants with ≥1 event, *n* (%)	Adjusted HR (95% CI)	P Value	E-value (point estimate)	E-value (lower CI)
Incarceration					0.001	1.74	1.40
No incarceration	671	956	257 (38.3)	1.0 (ref)			
Incarceration	687	3486	379 (55.2)	1.22 (1.09–1.37)			
Incarceration episodes					0.005		
No incarceration	671	956	257 (38.3)	1.0 (ref)			
1 episode	279	863	131 (46.9)	1.16 (1.02–1.33)		1.59	1.16
2 episodes	160	755	89 (55.6)	1.28 (1.10–1.47)		1.88	1.43
3 episodes	103	522	54 (52.4)	1.27 (1.04–1.54)		1.86	1.24
≥4 episodes	145	1346	105 (72.4)	1.33 (1.13–1.56)		1.99	1.51
Cumulative incarceration duration^a^					0.002		
0 days	729	1010	279 (38.3)	1.0 (ref)			
1–30 days	37	32	12 (32.4)	1.34 (1.07–1.68)		2.04	1.35
31–90 days	77	156	36 (46.8)	1.15 (0.92–1.44)		1.56	-
91–180 days	104	292	49 (47.1)	1.17 (0.98–1.41)		1.62	-
181–365 days	181	859	98 (54.1)	1.22 (1.03–1.43)		1.73	1.19
>365 days	230	2093	162 (70.4)	1.35 (1.16–1.57)		2.07	1.59

CI – Confidence interval.

^a^Cumulative incarceration duration represents the total number of days spent in custody accrued from study entry up to the beginning of each person-time interval where a participant was at risk for an event.

P values are test for heterogeneity.

Incident mental disorder diagnoses varied by sex and age. A higher proportion of females received a diagnosis compared to males (58.9% vs 45.5%), with differences observed for mood (14.7% vs 6.8%) and anxiety (14.1% vs 4.3%) disorders. Adolescents aged 10–13 at first offence had a higher incidence of mental disorders than those aged 14–17 (70.7% vs 41.7%).

### Association between incarceration and incident mental disorders

Among adolescents without pre-existing disorders, incarceration was associated with an increased risk of incident mental disorders (aHR: 1.22, 95% CI: 1.09–1.37; Table 4). This association showed a dose-response pattern by number of episodes, ranging from aHR 1.16 (95% CI: 1.02–1.33) for one episode to aHR 1.33 (95% CI: 1.13–1.56) for ≥4 episodes. By duration, highest aHRs were observed for brief (1–30 days: aHR 1.34, 95% CI: 1.07–1.68) and extended exposures (>365 days: aHR 1.35, 95% CI: 1.16–1.57). Full model parameters for all incarceration-related exposures are available in Supplemental Tables 5–7.

A qualitative interaction was observed between incarceration and socioeconomic disadvantage (Supplemental Table 8). Among adolescents with incarceration, those from the most disadvantaged areas had a higher risk of mental health disorders than those from the least disadvantaged areas (aHR: 1.21, 95% CI: 1.06–1.38). Conversely, among those without incarceration, adolescents from the most disadvantaged areas had a lower risk than those from the least disadvantaged areas (aHR: 0.81, 95% CI: 0.71–0.93). This interaction accounted for 31% of the risk among those with both risk factors (95% CI: 16%–45%).

### Sensitivity analyses

Several sensitivity analyses confirmed the robustness of the association between incarceration and incident mental disorders. Detailed parameter estimates for these analyses are provided in Supplemental Tables 9–11.

First, the association between incarceration and incident mental disorders persisted when excluding events that occurred within the first 6 months (aHR, 1.17; 95% CI, 1.02–1.34) and, separately, within the first year (aHR, 1.18; 95% CI, 1.03–1.35) after the start of incarceration.

Second, a stronger association between incarceration and the diagnosis of non-substance-related mental health disorders was noted (aHR, 1.45; 95% CI, 1.20–1.75), suggesting the main finding was likely not driven by substance-related events.

Third, E-value analyses assessed robustness to unmeasured confounding. For the primary association (aHR: 1.22), the E-value of 1.74 (lower CI: 1.40) indicates that unmeasured confounders would need to be associated with both incarceration and mental disorders by at least this magnitude to explain away the findings. E-values for sensitivity analyses ranged from 1.62 to 2.26 (Table 4), with the analysis excluding substance use disorders showing the greatest robustness to unmeasured confounding.

## Discussion

This study provides evidence for bidirectional associations between mental health disorders and incarceration among justice-involved adolescents. Our findings make two key contributions. First, showing that pre-existing mental disorders are associated with an increased incarceration risk provides a temporal mechanism explaining the over-representation of adolescents with mental health disorders in secure justice settings.^1^ While prior studies identified mental disorders as predictors of justice involvement,^9,20^ they could not definitively establish temporal sequence due to their inability to distinguish pre-existing from incident conditions. Second, showing that incarceration is associated with an increased risk of diagnosed mental disorders adds to evidence of the adverse consequences of incarceration in adolescence. Previous research has linked adolescent incarceration to poor quality of life,^21^ psychosocial problems,^22^ suicidality,^23^ and poor adult health outcomes.^24^

Our interaction analyses further demonstrate how individual, offence-related, and social factors modify risk. The synergistic interaction between pre-existing mental disorders and violent offences on incarceration risk provides quantitative evidence supporting specialized responses for violent youth with mental health needs.^25^ The differential impact of incarceration by socioeconomic disadvantage aligns with social determinants frameworks and evidence of socioeconomic gradients in stress vulnerability.^26,27^ Unexpectedly, among non-incarcerated adolescents, those from the most disadvantaged areas had lower risk of diagnosed mental disorders, contrasting with established associations between disadvantage and poor mental health.^28^ This apparent protective association likely reflects systemic barriers, as disadvantaged adolescents face documented obstacles to mental health service access,^29,30^ meaning only the most severe cases reach clinical attention. Unmeasured protective factors in this group may also contribute. Further research should disentangle these explanations.

The mechanisms underlying these associations remain incompletely understood. The observed relationship between incarceration and mental health diagnoses could reflect: (1) genuine iatrogenic effects of incarceration on mental health; (2) shared underlying vulnerabilities or third variables (such as genetic predisposition, early trauma, or family dysfunction) that independently increase risk for both mental disorders and justice involvement; (3) increased clinical detection during periods of system involvement rather than true incident disorders; or (4) residual confounding from unmeasured factors. Our sensitivity analyses excluding early post-incarceration diagnoses provide some evidence against pure detection bias but cannot definitively establish causal mechanisms.

### Limitations

Several important limitations constrain interpretation of these findings. First, our administrative data ascertainment captures only diagnosed mental health conditions severe enough to warrant healthcare contact, systematically under-representing the true burden of mental ill-health in this population. This may misclassify adolescents with undiagnosed conditions as disorder-free and cannot assess subclinical symptoms that may still have significant functional impact. Consequently, our findings likely underestimate the true burden of mental health problems and the magnitude of observed associations. Second, we cannot establish the precise timing of mental health symptom onset versus diagnosis, as administrative data capture healthcare encounters rather than disease onset.

Third, the exposure classification treats incarceration as a uniform experience, masking heterogeneity in facility conditions, therapeutic programming, exposure to violence, and access to mental health services. This prevents identification of which specific custodial elements drive adverse outcomes and limits guidance for targeted interventions. Fourth, despite covariate adjustment and E-value analyses indicating moderate robustness to confounding, unmeasured factors including genetic predisposition, trauma histories, family mental health status, and substance use patterns may still influence associations. Fifth, findings emerge from a single jurisdiction (NSW, Australia), limiting generalizability to systems with different legal frameworks, detention practices, or healthcare systems. Finally, while primary analyses tested a priori hypotheses, interaction findings should be considered exploratory.

## Conclusion

In this population-based cohort study of justice-involved adolescents, we provide evidence for a bidirectional association between mental disorders and incarceration. Our findings demonstrate that pre-existing mental disorders are associated with an increased risk of subsequent incarceration, while incarceration is, in turn, associated with an increased risk of incident mental disorder diagnoses among adolescents with no prior history of mental illness. These mutually reinforcing pathways might explain the profound over-representation of mental illness within youth justice systems. The evidence highlights the urgent need for a paradigm shift from a purely punitive model to an integrated health and justice framework, which would prioritize diversion for youth with mental health needs.

## Supplemental Material

Supplemental Material - Temporal relationships between incarceration and mental disorders among justice-involved adolescents: A population-based cohort study Supplemental Material for Temporal relationships between incarceration and mental disorders among justice-involved adolescents: A population-based cohort study by Emaediong I Akpanekpo, Azar Kariminia, Preeyaporn Srasuebkul, Julian N Trollor, John Kasinathan, David Greenberg, and Tony Butler in Australasian Psychiatry

## Data Availability

The data used in this study are not publicly available due to privacy and confidentiality restrictions. The data were obtained under strict data-sharing and ethics agreements that preclude public archiving.*

## References

[1] Beaudry G Yu R Långström N , et al. An updated systematic review and meta-regression analysis: mental disorders among adolescents in juvenile detention and correctional facilities. J Am Acad Child Adolesc Psychiatry 2021; 60: 46–60. 32035113 10.1016/j.jaac.2020.01.015 PMC8222965

[2] Akpanekpo EI Butler T Srasuebkul P , et al. Mental health disorders, adverse childhood experiences and accelerated reoffending among justice-involved youth in Australia: a longitudinal recurrent event analysis. Int J Law Psychiatr 2025; 101: 102099. 10.1016/j.ijlp.2025.102099 40286634

[3] Marr C Gaskin C Kasinathan J , et al. The prevalence of mental illness in young people in custody over time: a comparison of three surveys in New South Wales. Psychiatr Psychol Law 2024; 31: 235–253. 38628250 10.1080/13218719.2023.2192257 PMC11018085

[4] Abram KM Zwecker NA Welty LJ , et al. Comorbidity and continuity of psychiatric disorders in youth after detention: a prospective longitudinal study. JAMA Psychiatry 2015; 72: 84–93. 25426584 10.1001/jamapsychiatry.2014.1375 PMC4562696

[5] Teplin LA Potthoff LM Aaby DA , et al. Prevalence, comorbidity, and continuity of psychiatric disorders in a 15-year longitudinal study of youths involved in the juvenile justice system. JAMA Pediatr 2021; 175: e205807. 33818599 10.1001/jamapediatrics.2020.5807 PMC8022269

[6] Leckning B Condon JR Das SK , et al. Mental health-related hospitalisations associated with patterns of child protection and youth justice involvement during adolescence: a retrospective cohort study using linked administrative data from the Northern Territory of Australia. Child Youth Serv Rev 2023; 145: 106771.

[7] Browne CC Korobanova D Yee N , et al. Factors associated with postrelease healthcare and justice contact among prison entrants in New South Wales. Int J Forensic Ment Health 2025; 24: 143–154.

[8] Tedeschi F Horwitz SM Surko M , et al. Mental health service referral and treatment following screening and assessment in juvenile detention. J Am Acad Psychiatry Law 2024; 52: 460–469. 39393913 10.29158/JAAPL.240082-24

[9] Kasinathan J . Predictors of rapid reincarceration in mentally ill young offenders. Australas Psychiatry 2015; 23: 550–555. 26405242 10.1177/1039856215597532

[10] Allerton M Champion U Butler T , et al. 2003 NSW young people in custody health survey: key findings report. NSW Department of Juvenile Justice, 2003.

[11] Indig D Vecchiato C Haysom L , et al. 2009 young people in custody health survey: full report. Justice Health and Juvenile Justice, 2011.

[12] Justice Health & Forensic Mental Health Network, Juvenile Justice . 2015 young people in custody health survey: full report. Justice Health and Juvenile Justice, 2017.

[13] Kenny D Nelson P Butler T , et al. Young people on community orders health survey: key findings report. University of Sydney, 2006.

[14] Ogilvie JM Tzoumakis S Thompson C , et al. Psychiatric illness and the risk of reoffending: recurrent event analysis for an Australian birth cohort. BMC Psychiatry 2023; 23: 355. 37221485 10.1186/s12888-023-04839-0 PMC10207651

[15] Heard-Garris N Sacotte KA Winkelman TNA , et al. Association of childhood history of parental incarceration and juvenile justice involvement with mental health in early adulthood. JAMA Netw Open 2019; 2: e1910465. 31483468 10.1001/jamanetworkopen.2019.10465 PMC6727677

[16] Baglivio MT Wolff KT Piquero AR , et al. The relationship between adverse childhood experiences (ACE) and juvenile offending trajectories in a juvenile offender sample. J Crim Justice 2015; 43: 229–241.

[17] Doyle JJ . Child protection and child outcomes: measuring the effects of foster care. Am Econ Rev 2007; 97: 1583–1610. 29135212 10.1257/aer.97.5.1583

[18] Lee RD Fang X Luo F . The impact of parental incarceration on the physical and mental health of young adults. Pediatrics 2013; 131: e1188–1195. 23509174 10.1542/peds.2012-0627 PMC3608482

[19] Amorim LD Cai J . Modelling recurrent events: a tutorial for analysis in epidemiology. Int J Epidemiol 2015; 44: 324–333. 25501468 10.1093/ije/dyu222 PMC4339761

[20] Tolou-Shams M Folk JB Holloway ED , et al. Psychiatric and substance-related problems predict recidivism for first-time justice-involved youth. J Am Acad Psychiatry Law 2023; 51: 35–46. 36646452 10.29158/JAAPL.220028-21 PMC10019581

[21] de Ruigh EL Popma A Twisk JWR , et al. Predicting quality of life during and post detention in incarcerated juveniles. Qual Life Res 2019; 28: 1813–1823. 30875009 10.1007/s11136-019-02160-6 PMC6571096

[22] Thomas SE Abram KM Aaby D , et al. Incarceration and subsequent psychosocial outcomes: a 16-year longitudinal study of youth after detention. J Am Acad Child Adolesc Psychiatry 2025; S0890-8567(25): 00184. 10.1016/j.jaac.2025.04.004 PMC12353158 40220993

[23] Abram KM Choe JY Washburn JJ , et al. Suicidal ideation and behaviors among youths in juvenile detention. J Am Acad Child Adolesc Psychiatry 2008; 47: 291–300. 18216737 10.1097/CHI.0b013e318160b3ce PMC2945393

[24] Barnert ES Dudovitz R Nelson BB , et al. How does incarcerating young people affect their adult health outcomes? Pediatrics 2017; 139: e20162624. 28115536 10.1542/peds.2016-2624 PMC5260153

[25] Cocozza JJ Hartstone E Braff J . Mental health treatment of violent juveniles: an assessment of need. Crime Delinquen 1981; 27: 487–496.

[26] World Health Organization . Social determinants of mental health. World Health Organization, 2014. https://apps.who.int/iris/handle/10665/112828

[27] Hatch SL Dohrenwend BP . Distribution of traumatic and other stressful life events by race/ethnicity, gender, SES and age: a review of the research. Am J Community Psychol 2007; 40: 313–332. 17906927 10.1007/s10464-007-9134-z

[28] Reiss F . Socioeconomic inequalities and mental health problems in children and adolescents: a systematic review. Soc Sci Med 2013; 90: 24–31. 23746605 10.1016/j.socscimed.2013.04.026

[29] Kataoka SH Zhang L Wells KB . Unmet need for mental health care among U.S. Children: variation by ethnicity and insurance status. Aust J Pharm 2002; 159: 1548–1555. 10.1176/appi.ajp.159.9.1548 12202276

[30] Planey AM Smith SM Moore S , et al. Barriers and facilitators to mental health help-seeking among African American youth and their families: a systematic review study. Child Youth Serv Rev 2019; 101: 190–200.

