# Supplementary material for: Temporal relationships between incarceration and mental disorders among justice-involved adolescents: A population-based cohort study
Source: Australas Psychiatry. 2025 Oct 3;34(1):55–62. doi: 10.1177/10398562251383801 (PMC12819891; doi:10.1177/10398562251383801)
Supplement: Supplemental Material - Temporal relationships between incarceration and mental disorders among justice-involved adolescents: A population-based cohort study [file sj-pdf-1-apy-10.1177_10398562251383801.pdf]

Temporal Relationships Between Incarceration and Mental Disorders Among Justice-Involved Adolescents: A Population-Based Cohort Study

Supplementary Methods

Data Sources and Linkage

The study cohort was constructed by linking data from four cross-sectional health surveys of justice-involved adolescents with five longitudinal, population-level administrative data collections. Data linkage was performed by the NSW Centre for Health Record Linkage (CHeReL) using probabilistic matching algorithms. These algorithms use personal identifiers to link records for the same individual across different datasets, achieving a linkage rate of 99.9% for this cohort.

The source surveys, which employed a total population sampling method, included: the 2003 Young People in Custody Health Survey (YPiCHS), the 2003–2006 Young People on Community Orders Health Survey (YPoCOHS), the 2009 YPiCHS, and the 2015 YPiCHS.

The five linked administrative data collections were:

1. NSW Reoffending Database (ROD): Maintained by the NSW Bureau of Crime Statistics and Research (BOCSAR), this database contains comprehensive information on all criminal court appearances, convictions, and sentences for offences proven in NSW courts, including the type (eg, custody, community order) and duration of sentences.

2. NSW Admitted Patient Data Collection (APDC): This is a census of all inpatient admissions to NSW public and private hospitals. It includes dates of admission and separation and up to 50 diagnostic codes (coded using ICD-10-AM) for conditions treated during the admission.

3. NSW Mental Health Ambulatory Data Collection (MH-AMB): This database records all service contacts with community-based mental health services in NSW, including dates of service and associated diagnoses.

4. NSW Emergency Department Data Collection (EDDC): This collection captures information on all presentations to NSW emergency departments, including diagnoses. For this study, diagnoses coded using the Systematized Nomenclature of Medicine – Clinical Terms (SNOMED-CT) were mapped to their equivalent ICD-10-AM classifications.

5. NSW Registry of Births, Deaths, and Marriages (RBDM): This registry contains statewide data on all births and deaths, which was used to confirm dates of birth and ascertain parental death.

Supplementary Figure 1. Timeline of data source availability for the linked cohort study (1994-2022)

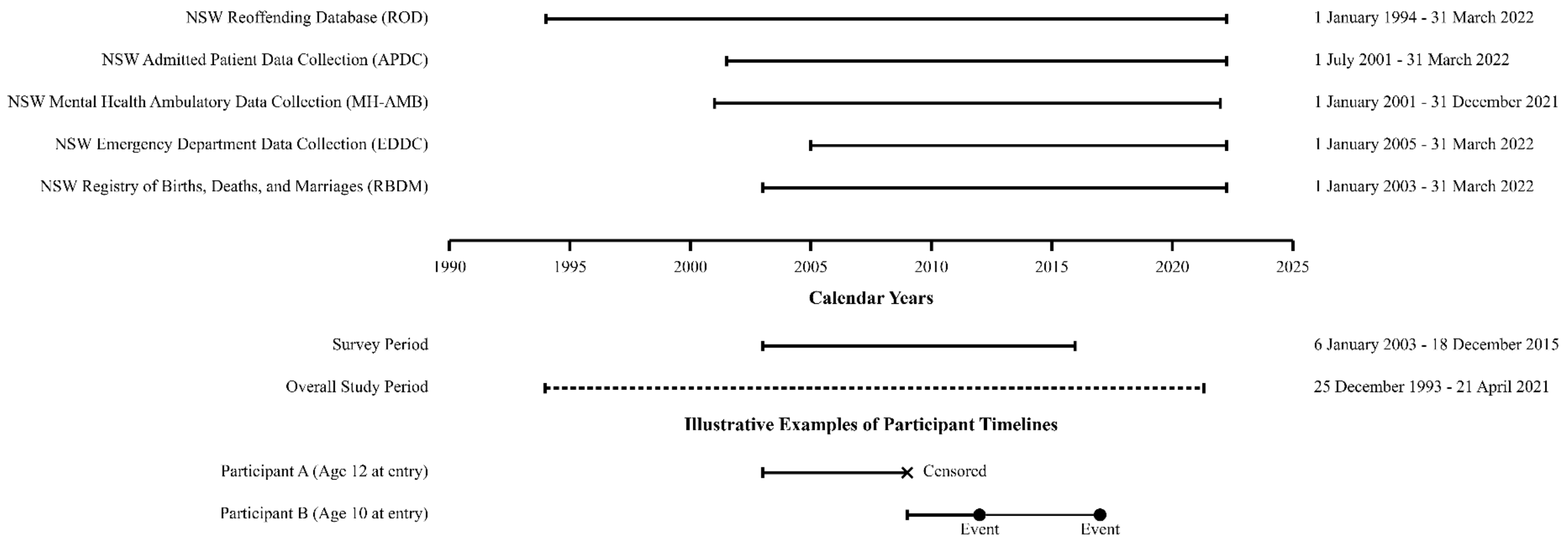

Participant Selection

The cohort selection process is outlined below (Supplementary Figure 2). The source health surveys identified 1,589 potential participants. Of these, probabilistic data linkage was successfully completed for 1,587 individuals. We then applied the study's inclusion criteria, requiring participants to be aged 10–17 years at the time of their first proven offence (index offence). We excluded 36 individuals: 3 were excluded due to a missing date of first offence, and 33 were excluded because their calculated age at first offence (accounting for leap years) was greater than 17 years. This resulted in a final study cohort of 1,551 adolescents.

Supplementary Figure 2. STROBE diagram illustrating participant inclusion and exclusion

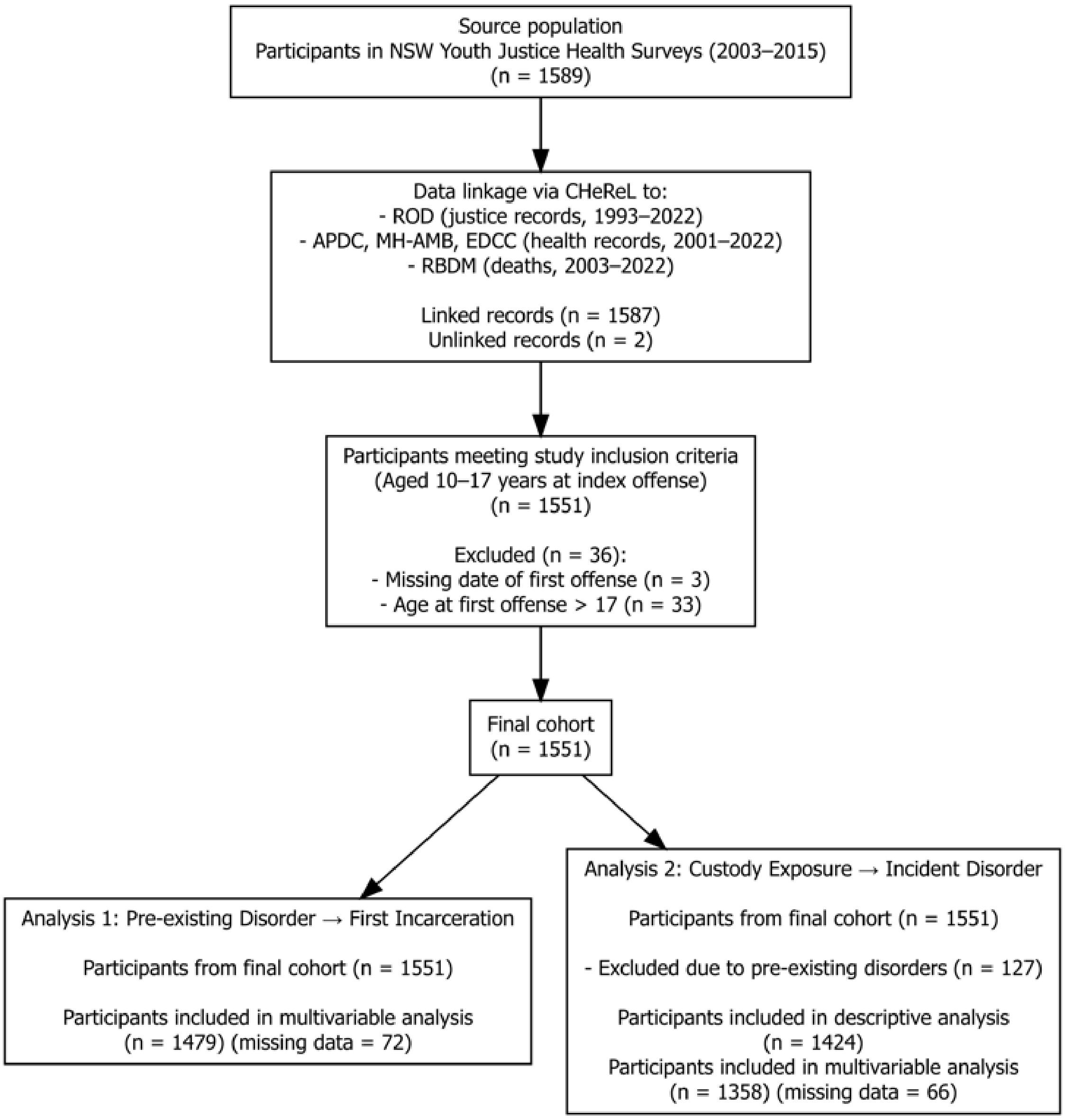

Variable Definitions

All exposures, outcomes, and covariates were defined and operationalised using the linked data collections. Detailed definitions for all variables used in the analyses are provided in Supplementary Table 1.

Supplementary Table 1: Definitions of Exposures, Outcomes, and Covariates

Variable Type	Variable Name	Definition and Operationalization	Data Source(s)
Exposure	Incarceration	The primary exposure, defined as incarceration in a NSW Youth Justice Centre. The start date of the exposure corresponded to the date of reception into the YJ centre, and the exposure period included any contiguous period of remand preceding court finalisation.<br>For the primary analysis of incident mental disorders (Objective 2), this was treated as a time-varying binary variable (0=not incarcerated, 1=incarcerated).	NSW Reoffending Database (ROD)
	Cumulative Incarceration Episodes	An alternative characterization of incarceration, treated as a time-varying categorical variable representing the total number of distinct incarceration episodes accrued by a participant from study entry. Categories were defined as 0, 1, 2, 3, or ≥4 episodes.	NSW Reoffending Database (ROD)
	Cumulative Incarceration Duration	An alternative characterization of the incarceration, treated as a time-varying categorical variable representing the total number of days spent in custody accrued from study entry. Categories were defined as 0, 1–30, 31–90, 91–180, 181–365, or >365 days.	NSW Reoffending Database (ROD)
Outcome	First Incarceration	The primary outcome for Objective 1. This was a binary variable indicating whether a participant experienced at least one episode of incarceration	NSW Reoffending Database

		during the follow-up period (from first proven offence to the 20th birthday).	(ROD)
	Incident Mental Disorder Diagnosis	The primary outcome for Objective 2. Defined as a new diagnosis of a mental disorder (see Table Supplementary Table 2 for codes) recorded during a distinct service episode in the linked health data collections. To prevent over-counting of related clinical encounters for a single illness episode, a minimum period of 30 days was required between diagnoses within the same diagnostic category.	NSW Admitted Patient Data Collection (APDC); NSW Mental Health Ambulatory Data Collection (MH-AMB); NSW Emergency Department Data Collection (EDDC)
Covariate	Pre-existing Mental Disorder	A binary variable indicating a history of a diagnosed mental disorder prior to the date of the participant's first proven offence (the index offence). A diagnosis was identified by the presence of a relevant ICD-10-AM code in any of the linked health datasets prior to the index date.	NSW Admitted Patient Data Collection (APDC); NSW Mental Health Ambulatory Data Collection (MH-AMB); NSW Emergency Department Data Collection (EDDC)
	Age at first offence	Participant's age in years at the date of their first proven offence. Treated as a continuous variable in all models.	NSW Reoffending Database (ROD)
	Sex	Participant's sex as recorded at the time of the baseline health survey. Coded as a binary variable (male/female).	Young People in Custody Health Surveys (YPiCHS); Young People on Community Orders Health Survey (YPoCOHS)
	Indigenous status	Participant's self-identified Indigenous status (Aboriginal and/or Torres Strait Islander peoples). Coded as a binary variable (Indigenous/Non-Indigenous).	YPiCHS; YPoCOHS
	Socio-economic	An area-level measure of relative disadvantage based on the participant's residential postcode at the time of the index offence. Assessed using the	NSW Reoffending Database

	Disadvantage	Index for Relative Socio-economic Disadvantage (IRSD) quartiles from the Australian Bureau of Statistics' Socio-Economic Indexes for Areas (SEIFA).	(ROD)
	Parental incarceration	A binary variable indicating whether the participant reported that one or both parents had ever been incarcerated.<br>For Objective 2, this was treated as a time-varying covariate.	YPiCHS; YPoCOHS
	Parental death	A binary variable indicating the death of one or both parents prior to the participant's 20th birthday.<br>For Objective 2, this was treated as a time-varying covariate.	YPiCHS; YPoCOHS
	Out-of-home care history	A binary variable indicating whether the participant reported ever having been in out-of-home care.<br>For Objective 2, this was treated as a time-varying covariate.	YPiCHS; YPoCOHS
	Head injury history	A binary variable indicating whether the participant self-reported a history of head injury resulting in loss of consciousness.<br>For Objective 2, this was treated as a time-varying covariate.	YPiCHS; YPoCOHS
	Index offence type	The most serious offence associated with the participant's first proven criminal charge. Offences were categorized as either 'Violent' or 'Non-violent' based on the Australian and New Zealand Standard Offence Classification (ANZSOC).	NSW Reoffending Database (ROD)
	Birth cohort	The participant's year of birth, grouped into three cohorts (1975–1984, 1985–1994, 1995–2004) to control for cohort effects.	NSW Reoffending Database (ROD)

Mental Disorder Classification

Supplementary Table 2 presents the International Statistical Classification of Diseases and Related Health Problems, Tenth Revision, Australian Modification (ICD-10-AM) codes used to define mental disorder diagnostic categories identified from the NSW Emergency Department Data Collection (EDDC), Mental Health Ambulatory Data Collection (MH-AMB), and Admitted Patients Data Collection (APDC).

Supplementary Table 2: Mental health disorder diagnostic categories and ICD 10 AM codes

Diagnostic group	Diagnostic category	ICD 10 AM codes
Psychosis	Schizophrenia, schizoaffective and other psychotic disorders	F20, F22, F23, F24, F25, F25.0, F25.1, F25.2, F25.8, F25.9, F28, F29
	Psychotic affective disorders	F30, F31, F32.3
	Psychotic disorders related to substance use	F10.5, F11.5, F12.5, F13.5, F14.5, F15.5, F15.50, F15.51, F15.59, F15.70, F16.5, F17.5, F18.5, F19.5, F19.7
Mood disorders	Major depressive disorder; recurrent depressive disorder; severe or persistent mood disorders; other mood disorders	F32.0, F32.1, F32.2, F32.8, F32.9, F33.0, F33.1, F33.4, F33.8, F33.9, F34, F38, F39
Anxiety disorders	Phobic anxiety disorders; acute stress reaction, post-traumatic stress disorder; adjustment disorders; dissociative and conversion disorders; somatoform disorders; other anxiety disorders	F40, F40.1, F40.2, F40.8, F40.9, F41, F42, F43.0, F43.1, F43.2, F43.8, F43.9, F41, F42, F44, F45, F48
Personality disorders	Clusters A, B, C and other personality disorders	F21, F60-F69
Substance use disorders	Mental and behavioural disorders due to use of alcohol and other substances	F10, F11, F12, F13, F14, F15, F16, F17, F18, F19 (excluding F10.5, F11.5, F12.5, F13.5, F14.5, F15.5, F16.5, F17.5, F18.5, F19.5, F19.7 which are classified under psychotic disorders)
Behavioural disorders	Behavioural and emotional disorders with onset usually occurring in childhood and adolescence	F90–F94; F98.7–F98.9 (Excluding F95, F98.0-F98.6)

ICD-10-AM: International Statistical Classification of Diseases and Related Health Problems, Tenth Revision, Australian Modification.

Statistical analysis

Analysis of Prevalent Mental Disorders and Incarceration Risk (Objective 1)

To investigate the association between pre-existing mental disorders and the subsequent risk of a first sentenced incarceration, we estimated Risk Ratios (RRs) and their 95% confidence intervals (CIs) using modified Poisson regression with a log link and robust error variances. This approach was chosen because it directly estimates the RR, which is more readily interpretable than an odds ratio, and provides a more conservative estimate when the outcome is common, as was the case in this study. The multivariable model was adjusted for the full set of a priori selected covariates, which were all treated as fixed at baseline and were retained in the final model regardless of their statistical significance to prevent bias from data-driven model selection. A second model was fitted that included a product term to formally test for statistical interaction on both the multiplicative and additive scales between pre-existing mental disorder status and the type of index offence (violent vs non-violent).

Analysis of Incarceration and Incident Mental Disorders (Objective 2)

To examine the association between time-varying incarceration exposure and the risk of incident recurrent mental disorder diagnoses, we analysed the subset of 1,424 adolescents with no diagnosed mental disorder prior to their first proven offence. We modelled the recurrent diagnoses using a Prentice–Williams–Peterson total-time (PWP-TT) model. This model is an extension of the standard Cox proportional hazards model specifically adapted for the analysis of recurrent event data. It was chosen because it appropriately handles time-varying covariates and allows the baseline hazard for a new diagnosis to differ for successive events within the same individual (eg, the risk of a second diagnosis may be different from the risk of a first), which is achieved by stratifying the analysis by event number.

Participants entered the at-risk population on the date of their first proven offence and were followed until they experienced a diagnosed mental health disorder (an event) or were censored on the day preceding their 20th birthday. Following a diagnosed event (the k-th event), the participant immediately became at risk for the subsequent (k+1-th) event and entered the corresponding (k+1)th event stratum, while the time scale continued to be measured from their original study entry date. To account for the non-independence of multiple events occurring within the same participant, cluster-robust standard errors were used. The proportional hazards assumption was assessed by testing for interactions with time; no violations were detected.

Sensitivity and Robustness Analyses

To assess the robustness of the findings linking incarceration exposure to incident mental disorders, several sensitivity analyses were conducted.

1. Lag-Time Analysis: To address the possibility of detection bias or reverse causation, whereby the increased clinical surveillance upon entering custody might lead to the rapid diagnosis of a pre-existing but previously undetected condition, we performed lag-time analyses. We repeated the

primary PWP-TT model but excluded any incident diagnoses that occurred within the first 6 months, and in a separate analysis, within the first 12 months, following the commencement of the first incarceration episode.

2. Exclusion of Substance Use Disorders: The relationship between substance use and justice system involvement is uniquely complex and often bidirectional. To test whether the primary finding was being disproportionately driven by this specific comorbidity, we conducted a sensitivity analysis that repeated the primary model after excluding all participants who received an incident substance use disorder diagnosis during the follow-up period.
3. E-value Calculation: To quantify the potential influence of unmeasured confounding, we calculated an E-value for the primary association and key sensitivity analyses. The E-value represents the minimum strength of association (on the risk ratio scale) that an unmeasured confounder would need to have with both the exposure (incarceration) and the outcome (mental disorder diagnosis), conditional on the measured covariates, to fully explain away the observed association. A larger E-value indicates greater robustness to potential unmeasured confounding.

**Supplementary Table 3. Full Results of Modified Poisson Regression Model for the Association Between Pre-existing Mental Disorders and First Sentenced Incarceration (Main Effects Model, N = 1479)**		
**Variable**	**Adjusted Risk Ratio (95% CI)**	**P value**
Prior mental disorder		0.002
No prior mental disorder	1.0 (ref)	
Prior mental disorder	1.26 (1.09–1.45)	
Index offence		0.294
Non-violent	1.0 (ref)	
Violent	1.05 (0.96–1.16)	
Sex		<0.001
Male	1.0 (ref)	
Female	1.68 (1.38–2.05)	
Age	0.88 (0.86–0.91)	<0.001
Indigenous status		<0.001
Non-Indigenous	1.0 (ref)	
Indigenous	1.44 (1.29–1.62)	
Parental incarceration	1.19 (1.08–1.31)	<0.001
Parental death	0.94 (0.80–1.11)	0.488
Out-of-home care	1.05 (0.94–1.18)	0.364
Head injury	0.96 (0.87–1.07)	0.496
Socioeconomic disadvantage		0.0004
Q1 (most disadvantaged)	1.0 (ref)	
Q2	0.92 (0.83–1.02)	
Q3	0.82 (0.72–0.93)	
Q4 (least disadvantaged)	0.67 (0.53–0.84)	

Abbreviations: CI, Confidence Interval; Ref, Reference category; Q, Quartile. Model adjusted for all variables listed. Socioeconomic disadvantage assessed using Index for Relative Socio-economic Disadvantage (IRSD) quartiles

**Supplementary Table 4. Interaction Between Pre-existing Mental Disorder and Violent Index Offence on the Risk of Sentenced Incarceration (N = 1479)**			
	Non-violent Index Offence	Violent Index Offence	RR (95% CI) for Violent vs. Non-violent within strata of Mental Disorder
No Pre-existing Mental Disorder	1.00 (ref)	1.01 (0.91–1.12); P = 0.880	1.01 (0.91–1.12); P = 0.842
Pre-existing Mental Disorder	1.02 (0.83–1.26); P = 0.823	1.62 (1.34–1.95); P < 0.001	1.48 (1.11–1.97); P = 0.008
RR (95% CI) for Pre-existing vs. No Pre-existing Mental Disorder within strata of Index Offence	1.02 (0.83–1.26); P = 0.837	1.59 (1.31–1.94); P < 0.001	—

Measure of interaction on additive scale: RERI (95% CI) = 0.60 (0.24–0.96); P = 0.001; Attributable proportion = 0.37 (95% CI: 0.19–0.54); P < 0.001
Measure of interaction on multiplicative scale: ratio of RRs (95% CI) = 1.57 (1.18–2.08); P = 0.002.
RRs are adjusted for sex, age, socio-economic disadvantage, Indigenous status, parental imprisonment, parental death, out-of-home care history, and head injury.

**Supplementary Table 5. Full Results of Prentice-Williams-Peterson Total Time (PWP-TT) Model for the Association Between Time-Varying Incarceration Exposure and Incident Mental Disorder Diagnosis (N = 1358)**		
**Variable**	**Adjusted Hazard Ratio (95% CI)**	**P value**
Incarceration status		0.001
No incarceration	1.0 (ref)	
Incarceration	1.22 (1.09–1.37)	
Age	1.13 (1.09–1.17)	<0.001
Sex		0.793
Male	1.0 (ref)	
Female	1.02 (0.89–1.16)	
Parental incarceration	1.10 (0.96–1.26)	0.163
Parental death	1.04 (0.88–1.23)	0.656
Head injury	0.97 (0.84–1.13)	0.728
Out-of-home care	0.93 (0.79–1.08)	0.336
Socioeconomic disadvantage		0.822
Q1 (most disadvantaged)	1.0 (ref)	
Q2	1.00 (0.90–1.12)	
Q3	0.98 (0.87–1.11)	
Q4 (least disadvantaged)	1.07 (0.90–1.26)	
Index offence		0.013
Non-violent	1.0 (ref)	
Violent	0.89 (0.81–0.98)	
Birth cohort		<0.001
1975–1984	1.0 (ref)	
1985–1994	2.30 (1.35–3.93)	
1995–2004	3.25 (1.88–5.63)	

Abbreviations: CI, Confidence Interval; Ref, Reference category; Q, Quartile. PWP-TT model adjusted for all variables listed. Incarceration status, parental incarceration history, parental death history, out-of-home care history, and head injury history were treated as time-dependent covariates.

**Supplementary Table 6. Full Results of Prentice-Williams-Peterson Total Time (PWP-TT) Model for the Association Between Number of Incarceration Episodes and Incident Mental Disorder Diagnosis (N = 1358)**		
**Variable**	**Adjusted Hazard Ratio (95% CI)**	**P value**
Cumulative Incarceration Episodes		0.005
No incarceration	1.0 (ref)	
1 episode	1.16 (1.02–1.33)	
2 episodes	1.28 (1.10–1.48)	
3 episodes	1.27 (1.04–1.54)	
4+ episodes	1.33 (1.13–1.56)	
Age	1.13 (1.10–1.17)	<0.001
Sex		0.899
Male	1.0 (ref)	
Female	1.01 (0.88–1.15)	
Parental incarceration	1.09 (0.95–1.25)	0.206
Parental death	1.04 (0.88–1.23)	0.619
Head injury	0.97 (0.84–1.12)	0.675
Out-of-home care	0.94 (0.80–1.10)	0.439
Socioeconomic disadvantage		0.768
Q1 (most disadvantaged)	1.0 (ref)	
Q2	1.01 (0.90–1.12)	
Q3	0.98 (0.87–1.11)	
Q4 (least disadvantaged)	1.08 (0.91–1.27)	
Index offence		0.009
Non-violent	1.0 (ref)	
Violent	0.88 (0.81–0.97)	
Birth cohort		<0.001
1975–1984	1.0 (ref)	
1985–1994	2.30 (1.35–3.93)	
1995–2004	3.27 (1.89–5.65)	

Abbreviations: CI, Confidence Interval; Ref, Reference category; Q, Quartile. PWP-TT model adjusted for all variables listed. Incarceration status, parental incarceration history, parental death history, out-of-home care history, and head injury history were treated as time-dependent covariates.

Supplementary Table 7. Full Results of Prentice-Williams-Peterson Total Time (PWP-TT) Model for the Association Between Cumulative Incarceration Duration and Incident Mental Disorder Diagnosis (N = 1358)

**Variable**	**Adjusted HR (95% CI)**	**P value**
Cumulative incarceration [illegible]		0.002
0 days	1.0 (ref)	
1–30 days	1.34 (1.07–1.68)	
31–90 days	1.15 (0.92–1.44)	
91–180 days	1.17 (0.98–1.41)	
181–365 days	1.22 (1.03–1.43)	
>365 days	1.35 (1.16–1.57)	
Age	1.13 (1.09–1.16)	<0.001
Sex		0.918
Male	1.0 (ref)	
Female	1.01 (0.88–1.15)	
Parental incarceration	1.09 (0.96–1.25)	0.189
Parental death	1.04 (0.88–1.23)	0.629
Head injury	0.98 (0.85–1.13)	0.740
Out-of-home care	0.92 (0.79–1.08)	0.314
Socioeconomic disadvantage		0.782
Q1 (most disadvantaged)	1.0 (ref)	
Q2	1.01 (0.91–1.13)	
Q3	0.99 (0.88–1.12)	
Q4 (least disadvantaged)	1.08 (0.92–1.28)	
Index offence		0.009
Non-violent	1.0 (ref)	
Violent	0.88 (0.81–0.97)	
Birth cohort		<0.001
1975–1984	1.0 (ref)	
1985–1994	2.26 (1.32–3.85)	
1995–2004	3.18 (1.84–5.51)	

Cumulative incarceration duration represents the total number of days spent in incarceration accrued from study entry up to the beginning of each person-time interval where a participant was at risk for an event.

Abbreviations: CI, Confidence Interval; Ref, Reference category; Q, Quartile. PWP-TT model adjusted for all variables listed. Incarceration status, parental incarceration history, parental death history, out-of-home care history, and head injury history were treated as time-dependent covariates.

Supplementary Table 8. Interaction Between Socio-economic Disadvantage and Incarceration Exposure on the Risk of Incident Mental Disorders (N = 1358)			
	**Least Socio-economic Disadvantage** †	**Most Socio-economic Disadvantage** †	aHR (95% CI) for Most vs. Least Socio-economic Disadvantage within strata of Incarceration
No incarceration	1.00 (ref)	0.83 (0.74–0.96); P = 0.008	0.81 (0.71–0.93); P = 0.002
Incarceration	0.99 (0.84–1.17); P = 0.939	1.20 (1.05–1.37); P = 0.006	1.21 (1.06–1.38); P = 0.004
aHR (95% CI) for Incarceration vs. No Incarceration within strata of Socio-economic Disadvantage	1.00 (0.83–1.19); P = 0.995	1.47 (1.28–1.69); P < 0.001	—

† Socio-economic disadvantage was recoded as a binary variable: "Least Socio-economic Disadvantage" (comprising the two least disadvantaged quartiles, Q3-Q4) and "Most Socio-economic Disadvantage" (comprising the two most disadvantaged quartiles, Q1-Q2), based on the Index for Relative Socio-economic Disadvantage (IRSD).
Measure of interaction on additive scale: RERI (95% CI) = 0.37 (0.20–0.54); P < 0.001; Attributable proportion = 0.31 (95% CI: 0.16–0.45); P < 0.001
Measure of interaction on multiplicative scale: ratio of RRs (95% CI) = 1.44 (1.17–1.71); P <0.001
aHRs are adjusted for sex, age, birth cohort, index offence, Indigenous status, parental imprisonment, parental death, out-of-home care history, and head injury.

**Supplementary Table 9: Sensitivity Analysis with 6-Month Exposure Lag: Parameter Estimates for All Covariates**		
**Variable**	**Adjusted Hazard Ratio (95% CI)**	**P value**
Incarceration status		0.021
No incarceration	1.0 (ref)	
Incarceration	1.17 (1.02–1.34)	
Age	1.14 (1.09–1.19)	<0.001
Sex		0.442
Male	1.0 (ref)	
Female	1.06 (0.92–1.21)	
Parental incarceration	1.12 (0.98–1.28)	0.095
Parental death	1.05 (0.88–1.25)	0.619
Head injury	0.98 (0.85–1.13)	0.807
Out-of-home care	0.89 (0.76–1.03)	0.124
Socioeconomic disadvantage		0.701
Q1 (most disadvantaged)	1.0 (ref)	
Q2	0.93 (0.83–1.05)	
Q3	0.96 (0.84–1.09)	
Q4 (least disadvantaged)	0.99 (0.83–1.20)	
Index offence		0.004
Non-violent	1.0 (ref)	
Violent	0.86 (0.78–0.95)	
Birth cohort		0.002
1975–1984	1.0 (ref)	
1985–1994	1.70 (0.99–2.94)	
1995–2004	2.11 (1.20–3.70)	

Abbreviations: CI, Confidence Interval; Ref, Reference category; Q, Quartile. PWP-TT model adjusted for all variables listed. Incarceration status, parental incarceration history, parental death history, out-of-home care history, and head injury history were treated as time-dependent covariates.

**Supplementary Table 10. Sensitivity Analysis with 1-year Exposure Lag: Parameter Estimates for All Covariates**		
**Variable**	**Adjusted Hazard Ratio (95% CI)**	**P value**
Incarceration status		0.015
No incarceration	1.0 (ref)	
Incarceration	1.18 (1.03–1.35)	
Age	1.14 (1.09–1.19)	<0.001
Sex		0.577
Male	1.0 (ref)	
Female	1.04 (0.90–1.20)	
Parental incarceration	1.11 (0.97–1.27)	0.113
Parental death	1.04 (0.87–1.26)	0.646
Head injury	0.98 (0.85–1.13)	0.779
Out-of-home care	0.89 (0.76–1.03)	0.126
Socioeconomic disadvantage		0.683
Q1 (most disadvantaged)	1.0 (ref)	
Q2	0.93 (0.83–1.05)	
Q3	0.97 (0.85–1.10)	
Q4 (least disadvantaged)	0.99 (0.82–1.19)	
Index offence		0.005
Non-violent	1.0 (ref)	
Violent	0.86 (0.78–0.96)	
Birth cohort		0.004
1975–1984	1.0 (ref)	
1985–1994	1.61 (0.91–2.84)	
1995–2004	1.97 (1.10–3.54)	

Abbreviations: CI, Confidence Interval; Ref, Reference category; Q, Quartile. PWP-TT model adjusted for all variables listed. Incarceration status, parental incarceration history, parental death history, out-of-home care history, and head injury history were treated as time-dependent covariates.

Supplementary Table 11. Sensitivity Analysis Excluding Participants with Substance-Related Disorders: Parameter Estimates for All Covariates		
**Variable**	**Adjusted Hazard Ratio (95% CI)**	**P value**
Incarceration status		<0.001
No incarceration	1.0 (ref)	
Incarceration	1.45 (1.20–1.75)	
Age	1.10 (1.05–1.16)	<0.001
Sex		0.422
Male	1.0 (ref)	
Female	0.89 (0.68–1.17)	
Parental incarceration	1.24 (1.03–1.50)	0.024
Parental death	1.12 (0.85–1.48)	0.420
Head injury	0.90 (0.73–1.09)	0.279
Out-of-home care	0.70 (0.57–0.87)	0.001
Socioeconomic disadvantage		0.016
Q1 (most disadvantaged)	1.0 (ref)	
Q2	1.23 (1.02–1.48)	
Q3	1.28 (1.05–1.55)	
Q4 (least disadvantaged)	1.42 (1.11–1.81)	
Index offence		0.077
Non-violent	1.0 (ref)	
Violent	0.87 (0.74–1.02)	
Birth cohort		<0.001
1975–1984	1.0 (ref)	
1985–1994	6.85 (1.68–27.98)	
1995–2004	10.19 (2.47–42.11)	

Abbreviations: CI, Confidence Interval; Ref, Reference category; Q, Quartile. PWP-TT model adjusted for all variables listed. Incarceration status, parental incarceration history, parental death history, out-of-home care history, and head injury history were treated as time-dependent covariates.
